# Factors associated with methicillin resistance in S*taphylococcus aureus*: a case–case–control analysis of AMR surveillance data

**DOI:** 10.1007/s10096-026-05544-y

**Published:** 2026-05-14

**Authors:** Godwin Pius Ohemu, Md. Kaisar Rahman, Yamima Tasnim, Samuel Ajulo, Babafela Awosile

**Affiliations:** School of Veterinary Medicine, Texas Tech University, 7671 Evans Dr, Amarillo, TX 79106 USA

**Keywords:** MRSA, MSSA, Antimicrobial resistance, Surveillance, Epidemiology, Case-case-control study

## Abstract

**Objective:**

This study examined clinical, demographic, and geographic factors associated with methicillin-resistant and methicillin-susceptible *Staphylococcus aureus* (MRSA and MSSA) isolation in a global surveillance dataset.

**Method:**

We analyzed Antimicrobial Testing Leadership and Surveillance (ATLAS) data (2004–2021) from 61 countries using a case-case-control design comparing MRSA and MSSA cases with *Enterococcus spp.* controls. Multivariable regression assessed factors associated with isolation. Model performance was assessed using VIF (< 2), Hosmer-Lemeshow tests (*p* > 0.05), and ROC-AUC (> 0.94).

**Results:**

We analyzed 155,482 isolates (54,599 MRSA, 63,101 MSSA, 37,782 Enterococcus). Models showed excellent discrimination (ROC-AUC: 0.945 for MRSA, 0.943 for MSSA). Substantial variation in isolation patterns was observed across countries. Compared with the United States, the relative MRSA odds were higher in Venezuela (OR = 1.66), Colombia (OR = 1.63), and Taiwan (OR = 1.57), while MSSA odds were higher in India, Kuwait, Brazil, Chile, Taiwan, and Israel. Compared with infants aged 0–2, children aged 3–12 and adolescents aged 13–18 years had higher odds of both MRSA and MSSA isolation. Relative to general surgery units, both MRSA and MSSA were more frequently isolated from the emergency and general medicine units. Compared with abdominal fluid samples, MRSA odds were highest in sputum (OR = 28.68), while MSSA showed a similar pattern in sputum (OR = 31.60), lower respiratory tract samples (OR = 26.26), abscesses (OR = 11.79), and wound samples (OR = 7.38). MRSA isolates also showed higher odds of levofloxacin co-resistance (OR = 11.19).

**Conclusion:**

This analysis describes variation in MRSA and MSSA characteristics across ATLAS settings. These findings may inform region-specific infection control strategies and highlight the need for continued global antimicrobial resistance surveillance.

**Supplementary Information:**

The online version contains supplementary material available at https://doi.org/10.1007/s10096-026-05544-y.

## Introduction

*Staphylococcus aureus* remains a leading pathogen globally, causing significant morbidity and mortality despite medical advances. This bacterium commonly colonizes skin and nasal mucosa, with approximately one in three individuals carrying it asymptomatically [1–3]. The challenge posed by this pathogen is further exacerbated by the emergence and global spread of methicillin-resistant *Staphylococcus aureus* (MRSA) [4]. Since the 1970s, MRSA has become endemic in healthcare facilities worldwide [5]. In 2019, the United States Centers for Disease Control and Prevention classified it as a “serious threat” requiring “prompt and sustained action” [6]. According to the Institute for Health Metrics and Evaluation, MRSA caused 121,000 deaths attributable to antimicrobial resistance (AMR) in 2019, making it the deadliest resistant pathogen worldwide [7].

While MRSA constitutes the major focus of clinical concerns, methicillin-susceptible *Staphylococcus aureus* (MSSA) also contributes significantly to the burden and has been implicated in numerous outbreaks [8, 9]. Recent surveillance from the United States and Europe has documented a paradoxical trend: declining MRSA incidence alongside rising MSSA infection [10, 11]. A population-based study from five U.S. states found that invasive MSSA infections were not only more common than MRSA across most demographic groups, but were also associated with comparable morbidity and mortality [11], highlighting the need to understand both pathogens comprehensively.

Understanding the epidemiology of MRSA and MSSA requires examination of multiple acquisition pathways. However, existing studies that examine the epidemiology of MRSA and MSSA focus on specific populations or individual countries [12, 13], limiting our understanding of global patterns and factors associated with isolation that may vary across healthcare systems and geographic regions. The Antimicrobial Testing Leadership and Surveillance (ATLAS) program provides a unique opportunity to examine these pathogens across diverse settings worldwide.

This study employed a case-case-control research design [14, 15], a methodology that allows simultaneous assessment of factors distinguishing resistant and susceptible organisms using a common comparison group. We aim to identify clinical, demographic, and geographic factors associated with MRSA and MSSA in a comprehensive multinational dataset spanning 61 countries over 18 years. By examining both MRSA and MSSA against a common control of *Enterococcus spp.* infections, we sought to characterize patterns that may inform targeted public health strategies and infection prevention efforts within a One Health framework. To our knowledge, this is the first global case-case-control analysis comparing MRSA and MSSA across > 60 countries using a standardized surveillance dataset.

## Methods

### Data source

The data used for this study were obtained from the Antimicrobial Testing Leadership and Surveillance (ATLAS) program (accessed at https://www.pfizer.com/science/focus-areas/anti-infectives/antimicrobial-surveillance on 15 June 2023), provided by the Vivli Center for Global Clinical Research Data (https://amr.vivli.org/). ATLAS is a global AMR surveillance initiative that collects bacterial isolates from participating medical centers for centralized susceptibility testing. The program employs standardized laboratory methods across all participating sites, with isolates tested at central reference laboratories using Clinical and Laboratory Standards Institute (CLSI) guidelines. AMR susceptibility testing followed CLSI criteria current at the time of testing. Although breakpoints for some antimicrobial agents changed over the study period, methicillin/oxacillin breakpoints remained stable, ensuring consistent MRSA/MSSA classification. Resistance variables were analyzed and reported as resistant vs. susceptible, reflecting clinically relevant co-resistant patterns. Missing data were minimal across core variables. Observations with missing values for any model variables were excluded, resulting in complete-case analysis. No substantial geographic pattern in missingness was observed. The findings of this analysis describe the patterns in clinical isolates submitted to the ATLAS surveillance program and may not reflect population-level disease burden or incidence rates. Based on ATLAS available documentation and protocol, the dataset used in this study includes non-duplicate isolates as unique ID was used for each observation in the dataset. This ensures each isolate represents an independent infection episode.

## Study design

We conducted a case-case-control study using a global antimicrobial surveillance dataset. A case-case-control study is a variant of the traditional case-control study design. It was formally introduced and validated for AMR research by Kaye et al. in a seminar paper in 2005 establishing the methodology as a standard approach. It is an epidemiological study design that compares two case groups (resistant pathogen vs. susceptible pathogen) against a common control group (different pathogen species) to identify factors specifically associated with resistance acquisition rather than general infection risk [14, 15]. This approach allowed us to identify factors associated with *Staphylococcus aureus* infections common to both MRSA and MSSA, and factors specifically associated with MRSA and MSSA to minimize bias inherent in traditional case-control studies of AMR by ensuring all study groups have similar healthcare acquisition patterns [15].

## Case and control definitions

We defined three study groups based on bacterial isolates as categorised and retrieved from ATLAS data: (1) MRSA cases: Methicillin-resistant *Staphylococcus aureus* isolates, defined by resistance to oxacillin or cefoxitin as classified by the ATLAS; (2) MSSA cases: Methicillin-susceptible *Staphylococcus aureus* isolates, defined by susceptibility to oxacillin or cefoxitin as classified by ATLAS; (3) Enterococcus control: *Enterococcus spp.* isolates from same surveillance system, same time frame, used as comparator for healthcare-associated bacterial pathogens with similar acquisition patterns but distinct antimicrobial resistance profile.

## Control group selection

*Enterococcus spp.* was selected as the control group because it represents a common Gram-positive healthcare-associated infection with similar ATLAS surveillance protocols, reducing differential ascertainment bias. The isolates were classified as *Enterococcus spp.* in the ATLAS database without species-level identification. The choice of enterococcus as control addresses the limitations of traditional case-control designs by ensuring cases and controls have comparable healthcare exposure, colonization pressure, and patient profile [14, 15]. This choice has some important implications for interpretation. As shown in Table 1, enterococcus differs from *S. aureus* in specimen distribution, with higher proportion from urine and abdominal fluid compared to MRSA and MSSA. This reflect known differences in pathogen tropism, as enterococcus is more commonly associated with urinary tract and intra-abdominal infections, while *S. aureus* more frequently causes skin/soft tissue and respiratory infection. We acknowledge that Enterococcus has distinct epidemiological characteristics, particularly higher prevalence in urinary tract and intra-abdominal infections, and different patient demographic patterns compared to *S. aureus*. These differences are addressed in the limitations section and must be considered when interpreting odds ratios as representing associations with pathogen isolation patterns rather than population-level risk factors for infection. Although sensitivity analyses using alternative control groups would help assess the impact of control selection, however, the study design (case-case-control study design) adopted for this study is well suitable for the nature of data used in this research project. The large sample size, minimal missingness, and strong model supports the robustness of our results and further validates the case-case-control design as appropriate for identifying factors associated with antimicrobial resistance while accounting for shared healthcare exposures [15].

**Table 1:** Characteristics of the Study Population by Case and Control Status

Characteristic	MRSA (*n* = 54,599)	MSSA (*n* = 63,101)	Enterococcus (*n* = 37,782)
**Year**,** median (IQR)**	2014 (2012–2016)	2015 (2012–2018)	2013 (2009–2018)
**Age group**,** n (%)**			
0–2 years	2,153 (4.0)	3,210 (5.1)	1,798 (4.8)
3–12 years	1,991 (3.7)	3,191 (5.1)	810 (2.2)
13–18 years	1,329 (2.5)	2,176 (3.5)	474 (1.3)
19–64 years	26,113 (48.3)	32,615 (52.3)	16,857 (45.1)
65–84 years	17,763 (32.8)	17,518 (28.1)	14,368 (38.5)
≥ 85 years	4,731 (8.7)	3,645 (5.8)	3,037 (8.1)
Missing	519 (1.0)	746 (1.2)	438 (1.2)
**Sex**,** n (%)**			
Female	22,039 (40.7)	26,231 (42.0)	17,100 (45.7)
Male	32,106 (59.3)	36,218 (58.0)	20,284 (54.3)
Missing	454 (0.8)	652 (1.0)	398 (1.1)
**Hospital specialty**,** n (%)**			
Surgery General	8,902 (16.3)	10,864 (17.2)	6,504 (17.2)
Surgery ICU	2,351 (4.3)	2,672 (4.2)	2,109 (5.6)
Medicine General	23,611 (43.2)	24,203 (38.4)	14,758 (39.1)
Medicine ICU	5,041 (9.2)	5,284 (8.4)	3,882 (10.3)
Pediatric General	2,194 (4.0)	3,489 (5.5)	1,233 (3.3)
Pediatric ICU	962 (1.8)	1,601 (2.5)	892 (2.4)
Emergency Room	5,790 (10.6)	6,792 (10.8)	2,793 (7.4)
General Unspecified ICU	842 (1.5)	720 (1.1)	390 (1.0)
Other†	4,906 (9.0)	7,476 (11.9)	5,221 (13.8)
**Specimen source**,** n (%)**			
Abdominal fluid	901 (1.7)	1,024 (1.6)	3,086 (8.2)
Abscess	5,621 (10.3)	5,063 (8.0)	696 (1.8)
Blood	7,725 (14.1)	13,085 (20.7)	11,616 (30.7)
Gastric abscess	2,612 (4.8)	3,299 (5.2)	2,239 (5.9)
Lower respiratory tract	5,172 (9.5)	6,525 (10.3)	818 (2.2)
Skin	2,310 (4.2)	3,188 (5.1)	981 (2.6)
Sputum	9,070 (16.6)	7,784 (12.3)	592 (1.6)
Ulcer	1,875 (3.4)	1,839 (2.9)	494 (1.3)
Urine	2,042 (3.7)	1,642 (2.6)	10,289 (27.2)
Wound	17,271 (31.6)	19,652 (31.1)	6,971 (18.5)

†Other includes Clinic/Office, Nursing Home/Rehab, and other unspecified settings.

IQR = interquartile range; ICU = intensive care unit.

## Variables 

### Outcome variables

 MRSA or MSSA isolation (yes/no), each compared to the enterococcus control group. 

**Explanatory variables included:**

- **Geographic**: Country of isolation (61 countries; the United States used as reference category).
- **Temporal**: Year of isolation (2004–2021, treated as a continuous variable to adjust for temporal trends).
- **Demographic**: Age group: 0–2, 3–12, 13–18, 19–64, 65–84, and ≥ 85 (reference: 0–2 years); sex: male and female (reference: female).
- Clinical setting: Hospital specialty unit including general surgery (reference), surgical intensive care unit (ICU), general medicine, medical ICU, general paediatrics, paediatric ICU, emergency, general unspecified, and others.
- Specimen source: Abdominal fluid (reference), abscess, blood, gastric abscess, lower respiratory tract, skin, sputum, ulcer, urine, and wound.
- AMR profiles: Resistance (vs. susceptibility) to seven critically important antimicrobials: levofloxacin, linezolid, tigecycline, vancomycin, daptomycin, teicoplanin, and erythromycin. These variables represent the AMR phenotype of the isolated organism based on standardized susceptibility testing, not prior patient antimicrobial exposure or treatment history. Therefore, these should be interpreted as co-resistance patterns characterising the pathogen rather than patient-level risk factors.

### Statistical analysis

Data management and cleaning were performed using the “tidyverse” package in R (4.4.2, R Foundation for Statistical Computing, Vienna, Austria; https://www.R-project.org/). Initial descriptive statistics were performed to summarize the data. We constructed two separate univariable and multivariable logistic regression models comparing MRSA and MSSA to enterococcus controls (Supplementary Table S1 and Table S2). Backward elimination based on Akaike Information Criterion (AIC) identified parsimonious models, with country and year retained regardless of significance. Model diagnostics included variance inflation factors (VIF) to assess multicollinearity, Hosmer-Lemeshow tests for calibration, and receiver operating characteristic area under the curve (ROC-AUC) for discrimination. Potential two-way interactions were examined between variables, retaining those with *p* < 0.05.

We calculated odds ratios (OR), 95% confidence intervals (CI), and *p*-values using Wald tests. Statistical significance was set at α = 0.05 (two-tailed). Variables significant for both MRSA and MSSA were interpreted as factors associated with *S. aureus* isolation in general; variables significant only for MRSA or only for MSSA were interpreted as factors specifically associated with resistant or susceptible strains, respectively. Country-level odds ratios were visualized using choropleth maps created with Datawrapper (https://www.datawrapper.de).

## Results

### Study population and model characteristics

Between 2004 and 2021, the ATLAS surveillance program collected 155,482 isolates meeting inclusion criteria for this analysis, comprising 54,599 MRSA isolates, 63,101 MSSA isolates, and 37,782 *Enterococcus spp.* control isolates from 61 countries (Table 1). After excluding observations with missing data on model variables, the final MRSA model included 52,150 observations (56.5% MRSA, 43.5% Enterococcus), and the MSSA model included 51,735 observations (54.9% MSSA, 45.1% Enterococcus).

Descriptive characteristics of this study population are presented in Table 1. The median collection year was 2014 (range: 2012–2016) for MRSA and 2015 (range: 2012–2018) for MSSA. MRSA was predominantly isolated from males (59.3%) and adults aged 19–64 years (48.3%), with the highest representation in the general medicine unit (43.2%) and wound specimens (31.6%). MSSA showed similar demographic patterns with 58.0% male isolates and 52.3% from adults aged 19–64 years, most commonly from general medicine units (38.4%) and wound specimens (31.1%).

### Model diagnostics

Both models demonstrated excellent fit and discriminatory ability. Multicollinearity assessment showed all VIF values were within acceptable limits (MRSA model range 1.0–1.21.0.21; MSSA model range: 1.00–1.13.00.13). The Hosmer-Lemeshow goodness-of-fit test showed statistically significant deviations from perfect fit (MRSA: χ²=46.20, *p* < 0.001; MSSA: χ²=22.44, *p* = 0.004), which is common in very large datasets (*n* > 50,000) where minor deviations become statistically detectable. However, both models showed excellent discrimination with ROC-AUC values of 0.945 for MRSA and 0.943 for MSSA, indicating strong predictive performance. There was no significant interactions.

### Temporal trends

After adjusting for all variables in the models, significant temporal trends were observed over the 18-year study period. The odds of both MRSA (OR = 0.75 per year, 95% CI: 0.74–0.76, *p* < 0.001) and MSSA (OR = 0.87 per year, 95% CI: 0.86–0.88, *p* < 0.001) isolation decreased over time relative to enterococcus. This observed year’s effect reflects relative odds within the ATLAS surveillance dataset, changes in ATLAS participation patterns over time, or a combination of these factors, and not population level prevalence or incidence trends.

### Geographic variation in pathogen isolation

**Fig. 1:**
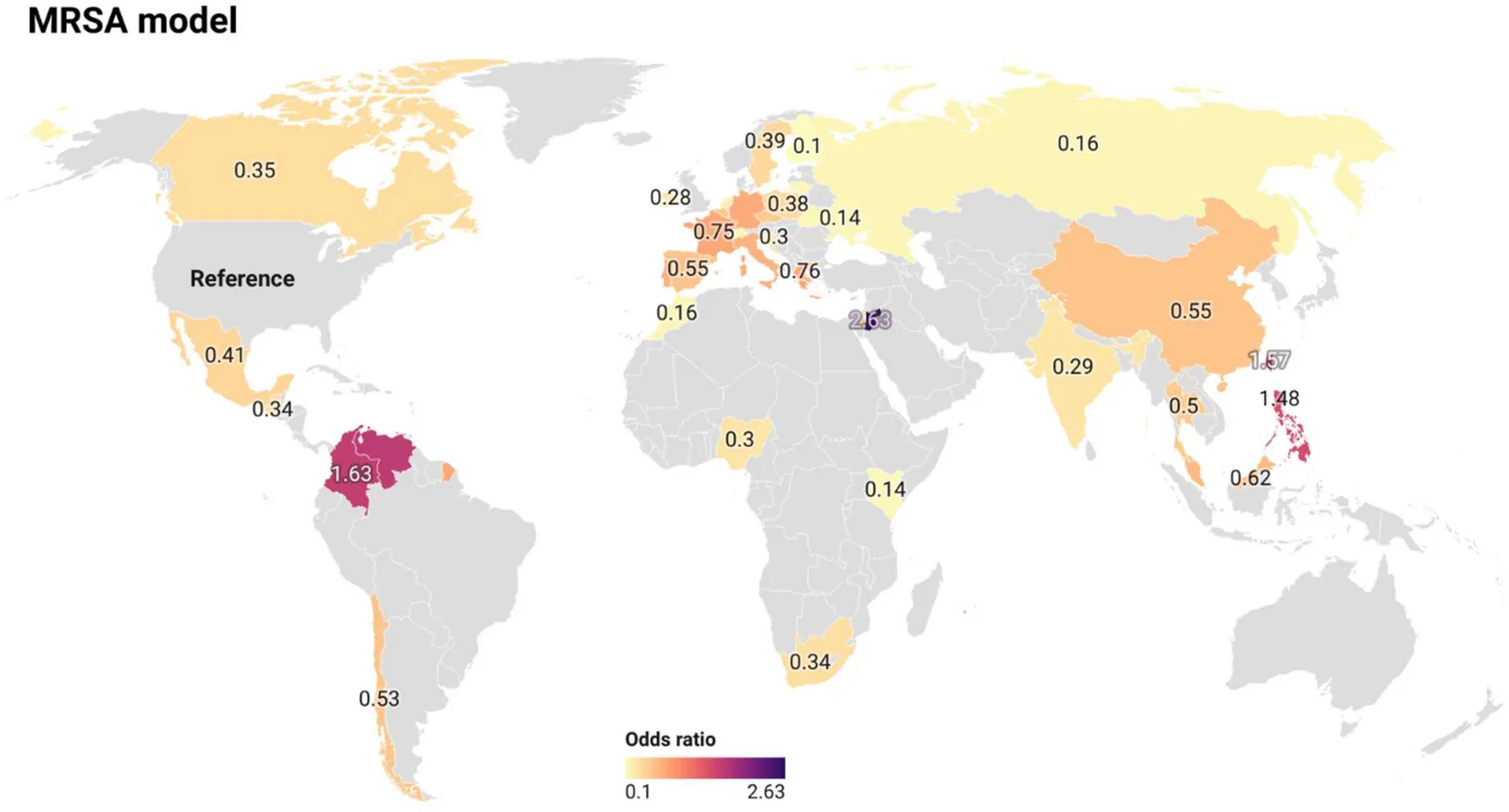
Country-level odds ratios from the multivariable logistic regression models of Methicillin-resistant *Staphylococcus aureus* (MRSA) (Reference: United States)

**Fig. 2:**
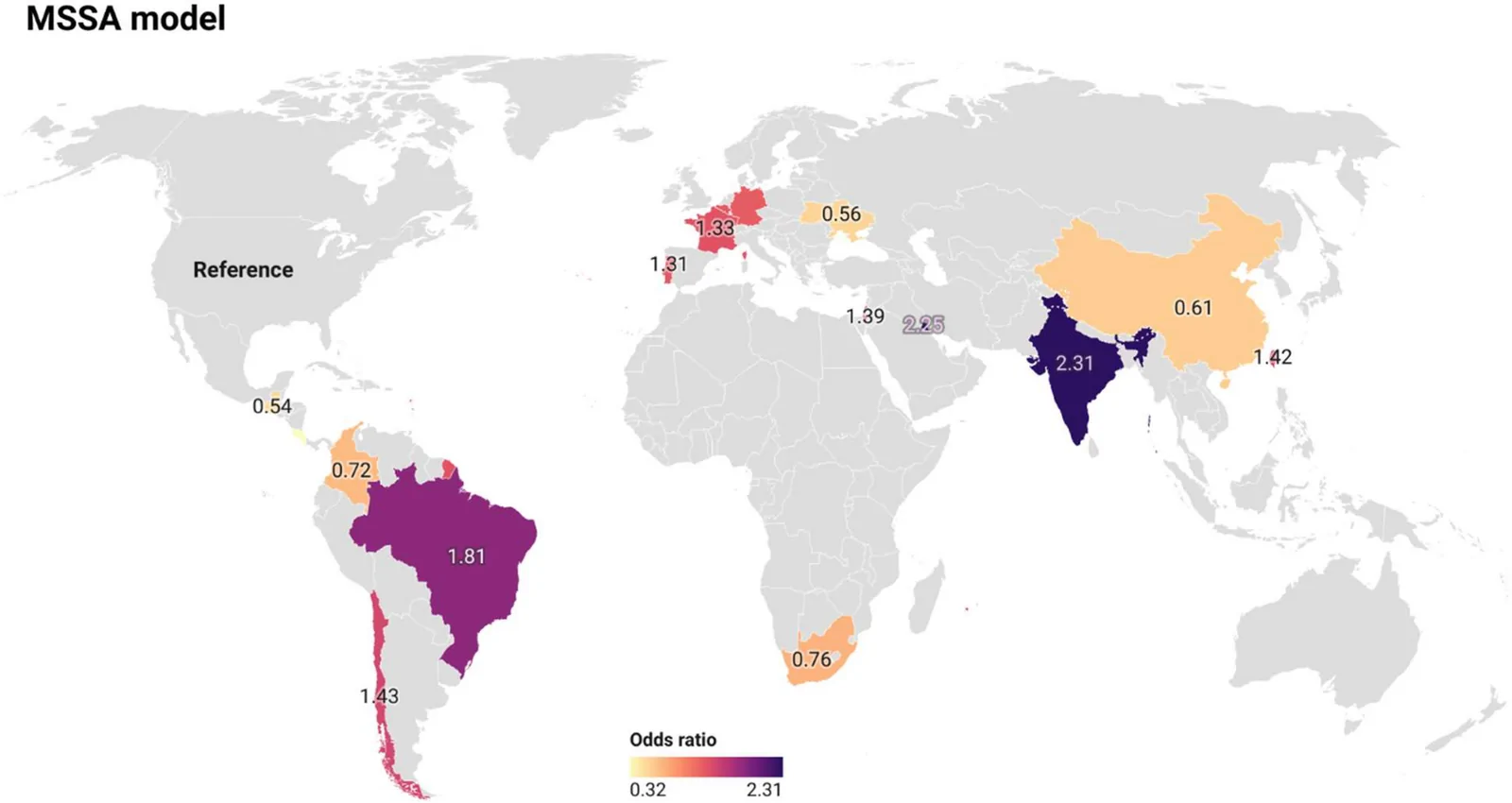
Country-level odds ratios from the multivariable logistic regression models of Methicillin susceptible *Staphylococcus aureus* (MSSA) (Reference: United States)

A total of 61 countries were included in this analysis, with the United States serving as the reference category for geographic comparisons. Substantial variation in the odds of MRSA and MSSA isolation was observed across countries (Figs. 1–2, Supplementary Table S2). These geographic differences should be interpreted with caution as they may reflect variations in ATLAS surveillance participation, sampling strategies, laboratory practices, and patient populations across countries, rather than true epidemiological differences. The number of contributing centers and isolates varied substantially by country (Supplementary Table S3), with some countries represented by a single center and limited sample sizes, contributing to wide confidence intervals in country-specific estimates. We acknowledge the possibility of clustering and sparse-data instability as some countries might only contribute data only during a specific year which will affect temporal comparability of geographic patterns.

For MRSA (Fig. 1), compared to the United States, the odds of MRSA isolation (relative to enterococcus) were significantly higher in Jordan (OR = 2.63, 95% CI: 1.19–6.19, *p* = 0.02), Venezuela (OR = 1.66, 95% CI: 1.29–2.14, *p* < 0.001), Colombia (OR = 1.63, 95% CI: 1.29–2.07, *p* < 0.001), Taiwan (OR = 1.57, 95% CI: 1.24–2.00.24.00, *p* < 0.001), and Philippines (OR = 1.48, 95% CI:1.17–1.90, *p* = 0.001). Conversely, the odds of MRSA isolation were significantly lower in 33 countries including Finland (OR = 0.10, 95% CI: 0.02–0.42, *p* = 0.005), Ukraine (OR = 0.14, 95% CI: 0.08–0.25, *P* < 0.001), Kenya (OR = 0.14, 95% CI: 0.07–0.34, *p* < 0.001), and multiple European nations (Complete results in Supplementary Table S2).

For MSSA (Fig. 2), the odds of MSSA isolation were significantly higher in India (OR = 2.31, 95% CI: 1.79–2.99, *p* < 0.001), Kuwait (OR = 2.20, 95% CI: 1.71–2.96, *p* < 0.001), Brazil (OR = 1.81, 95% CI: 1.46–2.25, *p* < 0.001), Chile (OR = 1.43, 95% CI: 1.09–1.87. *p* = 0.01), Taiwan (OR = 1.42, 95% CI: 1.11–1.84, *p* = 0.06), Israel (OR = 1.39, 95% CI: 1.09–1.77, *p* = 0.008), France (OR = 1.33, 95% CI: 1.11–1.60, *p* = 0.002), Portugal (OR = 1.13, 95% CI: 1.05–1.64, *p* = 0.019), Belgium (OR = 1.29, 95% CI: 1.06–1.59, *p* = 0.013), and Germany (OR = 1.27, 95% CI: 1.05–1.54, *p* = 0.015). The odds of MSSA isolation were significantly lower in China (OR = 0.61, 95% CI: 0.52–0.72, *p* < 0.001), Colombia (OR = 0.72, 95% CI: 0.56–0.93, *p* = 0.012), Costa Rica (OR = 0.32, 95% CI: 0.17–0.58, *p* < 0.001), Guatemala (OR = 0.54, 95% CI: 0.38–0.78, *p* = 0.001), South Africa (OR = 0.76, 95% CI: 0.60–0.98, *p* = 0.032), and Ukraine (OR = 0.56, 95% CI: 0.37–0.88, *p* = 0.009) (complete results in Supplementary Table S2).

### Demographic characteristics

#### Sex

No significant association was observed between sex and MRSA isolation (OR = 1.05, 95% CI: 0.99–1.22, *p* = 0.11) or MSSA isolation (OR = 0.02, 95% CI: 0.96–1.09, *p* = 0.49) (Table 2).

**Table 2:** Factors Associated with MRSA and MSSA Isolation: Multivariable Logistic Regression Results

Variable	MRSA Model	MSSA Model
	OR (95% CI)	OR (95% CI)
**Year (continuous)**	0.75 (0.74–0.76)***	0.87 (0.86–0.88)***
**Sex**		
Female (ref)	1.00	1.00
Male	1.05 (0.99–1.12)	1.02 (0.96–1.09)
**Age group**		
0–2 years (ref)	1.00	1.00
3–12 years	1.59 (1.25–2.03)***	2.01 (1.60–2.54)***
13–18 years	1.41 (1.05–1.89)*	2.27 (1.71–3.03)***
19–64 years	0.74 (0.60–0.93)**	1.17 (0.94–1.45)
65–84 years	0.52 (0.42–0.65)***	0.81 (0.65–1.01)
≥ 85 years	0.59 (0.46–0.76)***	0.91 (0.71–1.16)
**Hospital specialty**		
Surgery General (ref)	1.00	1.00
Surgery ICU	0.78 (0.66–0.92)**	0.74 (0.63–0.87)***
Medicine General	1.32 (1.21–1.45)***	1.29 (1.18–1.41)***
Medicine ICU	1.12 (0.97–1.29)	0.98 (0.85–1.13)
Pediatric General	1.07 (0.84–1.36)	1.23 (0.97–1.56)
Pediatric ICU	0.87 (0.66–1.16)	0.82 (0.62–1.08)
Emergency Room	1.60 (1.41–1.81)***	1.67 (1.47–1.89)***
General Unspecified ICU	0.96 (0.77–1.21)	0.78 (0.61–0.99)*
Other	1.13 (0.96–1.32)	1.29 (1.11–1.51)***
**Specimen source**		
Abdominal fluid (ref)	1.00	1.00
Abscess	13.93 (11.59–16.76)***	11.79 (9.80–14.21.80.21)***
Blood	3.42 (2.90–4.05)***	3.83 (3.24–4.53)***
Gastric abscess	2.30 (1.86–2.85)***	2.34 (1.89–2.89)***
Lower respiratory tract	21.14 (16.90–26.48.90.48)***	26.26 (20.96–33.01)***
Skin	7.16 (5.68–9.05)***	6.12 (4.87–7.70)***
Sputum	28.68 (23.31–35.28)***	31.60 (25.65–39.04)***
Ulcer	7.32 (5.83–9.21)***	7.97 (6.34–10.04)***
Urine	0.51 (0.43–0.61)***	0.46 (0.39–0.55)***
Wound	8.26 (7.03–9.71)***	7.38 (6.27–8.68)***

*p < 0.05; **p < 0.01; ***p < 0.001

OR = odds ratio; CI = confidence interval; ICU = intensive care unit; ref = reference category

#### Age

Compared to infants aged 0–2 years, the odds of MRSA isolation were significantly higher in children aged 3–12 years (OR = 1.59, 95% CI: 1.25–2.03, *p* < 0.001) and adolescents aged 13–18 years (OR = 1.41, 95% CI:1.05–1.89, *p* = 0.023). The odds were significantly lower in adults aged 19-64years (OR = 0.74, 95% CI: 0.60–0.93, *p* = 0.008), 65-84years (OR = 0.52, 95% CI: 0.42-0.42.65Z, *p* < 0.001), and > 85 years (OR = 0.59, 95% CI: 0.46–0.76, *p* < 0.001).

For MRSA, the odds of isolation were significantly higher in children aged 3 − 2 years (OR = 2.01, 95% CI: 1.60–2.54, *p* < 0.001) and adolescents aged 13–18 years (OR = 2.27, 95% CI: 1.71–3.03, *p* < 0.001) compared to infants aged 0–2 years. No significant differences were observed for other adult age groups (Table 2).

Country-level results are presented in Figs. 1–2 and Supplementary Table S2 and S3. Univariable result and associated crude odds ratios, and the outcomes are presented in Supplementary Table S1.

### Clinical settings

MRSA and MSSA isolation patterns varied significantly across hospital speciality units (Table 2). Compared to general surgery units, the odds of MRSA isolation were significantly higher in emergency departments (OR = 1.60, 95% CI: 1.41–1.81, *p* < 0.001) and general medicine units (OR = 1.32, 95% CI: 1.21–1.45, *p* < 0.001), while they were significantly lower in surgical ICU units (OR = 0.78, 95% CI: 0.66–0.92, *p* = 0.003).

For MSSA, the odds of isolation were similarly elevated in emergency departments (OR = 1.67, 95% CI: 1.47–1.89, *p* < 0.001), general medicine units (OR = 1.29, 95% CI: 1.18–1.41, *p* < 0.001), and other specialty units (OR = 1.29, 95% CI: 1.11–1.51, *p* < 0.001) compared to general surgery. The odds were significantly lower in the general unspecified ICU (OR = 0.78, 95% CI: 0.61–0.99, *p* = 0.041) and surgical ICU (OR = 0.74, 95% CI: 0.63–0.87, *p* < 0.001).

### Specimen source

Both MRSA and MSSA showed substantially higher odds of isolation for most anatomical sites compared to abdominal fluid samples (Table 2). For MRSA, the odds of isolation were highest in sputum specimens (OR = 28.68, 95% CI: 23.31–35.28, *p* < 0.001), followed by lower respiratory tract samples (OR = 21.14, 95% CI: 16.90–26.48.90.48, *p* < 0.001), abscesses (OR = 13.93, 95% CI:11.59–16.76, *p* < 0.001), wounds (OR = 8.26, 95% CI: 7.03–9.71, *p* < 0.001), ulcers (OR = 7.32, 95% CI: 5.83–9.21, *p* < 0.001), skin (OR = 7.16, 95% CI: 5.68–9.05, *p* < 0.001), blood (OR = 3.42, 95% CI: 2.90–4.05, *p* < 0.001), and gastric abscesses (OR = 2.30, 95% CI: 1.86–2.85, *p* < 0.001). Conversely, the odds of MRSA isolation from urine were significantly lower than from abdominal fluid (OR = 0.51, 95% CI: 0.43–0.61, *p* < 0.001).

Similar patterns were observed for MSSA, with highest odd of isolation in sputum (OR = 31.60, 95% CI: 25.65–39.04, *p* < 0.001), lower respiratory tract (OR = 26.26. 95% CI: 20.96–33.01, *p* < 0.001), abscesses (OR = 11.79, 95% CI: 9.80–14.21.80.21, *p* < 0.001), ulcers (OR = 7.97, 95% CI: 6.34–10.04, *p* < 0.001), wounds (OR = 7.38, 95% CI: 6.27–8.68, *p* < 0.001), skin (OR = 6.12, 95% CI: 4.87–7.70, *p* < 0.001), blood (OR = 3.83, 95% CI: 3.24–4.53, *p* < 0.001), and gastric abscesses (OR = 2.34, 95% CI: 1.89–2.89, *p* < 0.001). MSSA isolation from urine was also significantly lower than from abdominal fluid (OR = 0.46, 95% CI: 0.39–0.55, *p* < 0.001).

### Antimicrobial co-resistance patterns

The antimicrobial variables in this analysis represent resistance phenotypes of the isolated organisms based on ATLAS standardized laboratory susceptibility testing, not prior patient exposure to these agents or treatment received. Therefore, these results characterize co-resistance patterns rather than identifying antimicrobial exposure as a risk factor for infection (Table 3).

**Table 3:** Antimicrobial Co-Resistance Patterns in MRSA and MSSA Isolates

Antimicrobial	MRSA Model	MSSA Model
	OR (95% CI)	OR (95% CI)
Levofloxacin	11.19 (10.35–12.11)***	0.47 (0.43–0.52)***
Linezolid	0.016 (0.008–0.030)***	0.022 (0.006–0.061)***
Tigecycline	0.41 (0.32–0.53)***	0.29 (0.21–0.40)***
Vancomycin	0.004 (0.002–0.010)***	< 0.001***
Daptomycin	0.002 (0.002–0.003)***	0.006 (0.005–0.008)***
Teicoplanin	0.35 (0.12–0.97)*	0.13 (0.02–0.74)*
Erythromycin	0.24 (0.22–0.26)***	0.075 (0.070–0.081)***

*p < 0.05; ***p < 0.001

OR = odds ratio; CI = confidence interval

ORs represent the odds of resistance (vs. susceptibility) to each antimicrobial in MRSA or MSSA isolates compared to enterococcus controls. These reflect organism co-resistance patterns, not patient-level antimicrobial exposures (more details in supplementary table S2)

The most striking findings were the markedly higher odds of levofloxacin resistance in MRSA isolates compared to controls (OR = 11.19, 95% CI: 10.35–12.11, *p* < 0.001), reflecting the well-documented co-occurrence of methicillin and fluoroquinolone resistance in *S. aureus* strains. For other antimicrobials tested, MRSA showed significantly lower odds of resistance to linezolid (OR = 0.016, 95% CI: 0.008–0.030, *p* < 0.001), tigecycline (OR = 0.41 C, 95% CI: 0.32–0.53, *p* < 0.001), vancomycin (OR = 0.004, 95% CI: 0.002–0.010, *p* < 0.001), daptomycin (OR = 0.002, 95% CI: 0.002–0.003, *p* < 0.001), teicoplanin (OR = 0.35, 95% CI: 0.12–0.97, *p* = 0.043), and erythromycin (OR = 0.24%, 95% CI, 0.22–0.26, *p* < 0.001) compared to enterococcus controls.

MSSA isolates showed similar patterns, with lower odds of resistance to levofloxacin (OR = 0.47, 95% CI: 0.43–0.52, *p* < 0.001), linezolid (OR = 0.022, 95% CI: 0.006–0.061, *p* < 0.001), tigecycline (OR = 0.29, 95% CI:0.21–0.40, *p* < 0.001), vancomycin (OR < 0.001, *p* < 0.001), daptomycin (OR = 0.006, 95% CI: 0.005–0.008, *p* < 0.001), teicoplanin (OR = 0.13, 95% CI: 0.02–0.74, *p* = 0.029), and erythromycin (OR = 0.075, 95% CI: 0.070–0.081, *p* < 0.001) compared to enterococcus controls.

## Discussion

This analysis of *S. aureus* and *Enterococcus spp*. from 61 countries between 2004 and 2021 identified substantial geographic, temporal, demographic, and clinical variation in factors associated with MRSA and MSSA isolation. Compared to the United States, several countries showed significantly elevated odds of MRSA isolation including Jordan (OR = 2.63), Venezuela (OR = 1.66), and Colombia (OR = 1.63), while MSSA isolation was higher in India (OR = 2.31), Kuwait (OR = 2.20), and Brazil (OR = 1.81). Relative to enterococcus controls, both MRSA and MSSA showed decreasing odds of isolation over the study period (OR = 0.75 and 0.87 per year respectively, both *p* < 0.001). Children aged 3–18 years had higher odds of both MRSA and MSSA isolation compared to infants, while older adults showed lower odds, likely reflecting the high prevalence of enterococcus infection in elderly patients. Emergency departments and general medicine units showed elevated odds of staphylococcal isolation compared to surgical units. Respiratory specimens such as sputum, lower respiratory tract had markedly higher odds of *S. aureus* isolation, while urine specimens showed lower odds. MRSA demonstrated strong co-resistance with levofloxacin (OR = 11.19) but low resistance to glycopeptides, linezolid, and daptomycin.

Before interpreting the findings of this analysis in detail, we acknowledge several limitations of our study. First, ATLAS is a hospital-based laboratory surveillance system, not a population-based study. Participating centers are predominantly large academic hospitals in high-income countries, with limited representation from the global south, smaller community hospitals, and resourced limited settings. Participating sites are not randomly selected and may not represent the broader healthcare landscape within each country. This sampling framework means our findings reflect MRSA and MSSA patterns in participating surveillance hospitals rather than population-based infection epidemiology. To address this potential bias, we included country as a covariate in all models, noted standardized CLSI-based susceptibility testing as reported by ATLAS across all sites, and interpreted findings as surveillance-based association rather than population-level risk factor. Additionally, country-level estimates reflect participating center characteristics rather than nationally representative samples, with some countries represented by few centers and limited sizes contributing to wider confidence intervals. Second, our choice of enterococcus as the control group may introduce interpretation challenges as enterococcus have distinct epidemiological characteristics compared to *S. aureus*. We acknowledged this limitation by framing our results as factors associated with pathogen isolation patterns rather than disease risk factors, and we accounted for specimen source differences in multivariable models.

Third, the temporal trends reflect relative changes in pathogen proportions within the surveillance network, not absolute population-level incidence or prevalence. The requirement for minimum annual isolate submission may distort temporal patterns, as centers experiencing declining infection rates require longer collection periods to meet quotas. Fourth, the antimicrobial resistance variables used in this analysis represent organism phenotypes rather than patient exposures and this may limit causal inference about antimicrobial use as a risk factor. To address this, we characterized our findings as co-resistance patterns. Finally, ATLAS lacks detailed clinical data including comorbidities, prior healthcare exposures, and antimicrobial use history. We addressed this challenge by including available demographic and clinical variables and by using a large sample size (> 50,000 isolates) to enhance statistical power and enable detection of robust association despite unmeasured confounding.

Despite these limitations, our study provides valuable insights into the global epidemiology of *S. aureus*. The dataset for this study encompasses 18 years of surveillance across 61 countries with ATLAS standardized laboratory methods, which provided broad geographic and temporal representation. The case-case-control design of this study enabled the simultaneous evaluation of factors associated with MRSA and MSSA, thereby allowing identification of resistance-specifics versus general staphylococcal patterns. The large sample size provided adequate statistical power to detect associations even for moderately represented countries. Model diagnostics demonstrated excellent discrimination (ROC-AUC 0.945 for MRSA, 0.943 for MSSA) and acceptable multicollinearity (VIF 1.00–1.21.00.21), supporting the validity of our analytical approach.

The substantial geographic differences in MRSA and MSSA patterns reflects the difference in how common these infections are in different countries, or the difference in how ATLAS surveillance operates in each location. The elevated MRSA odds in Latin American countries such as Venezuela and Colombia aligns with previous reports of high MRSA prevalence in this region [16, 17], while the low MRSA odds in Nordic countries such as Finland (OR = 0.10) are consistent with successful IPC programs in this region [18]. The higher MSSA odds in India and Kuwait may indicate higher overall *S. aureus* burden, better detection practices for susceptible organisms, or different enterococcus epidemiology. Taiwan’s elevated odds for both MRSA and MSSA suggest overall higher staphylococcal isolation rates, potentially reflecting endemic disease or facility-specific patient population. The geographic findings of this study should be interpreted with caution as country-level estimates reflect participating ATLAS centers rather than nationally representative samples and may be influenced by clustering and uneven data availability. Additionally, interpreting these findings must consider ATLAS sampling differences across countries. Facilities with different patient populations, types of care, and specimen collection practices may show obvious geographic differences reflecting surveillance characteristics rather than true epidemiological variation. These findings have implications beyond clinical settings, reflecting broader antimicrobial selection pressures across human, animal, and environmental interfaces.

The decreasing temporal patterns observed for MRSA and MSSA isolation relative to enterococcus over 18 years in our study, and with a steeper decline for MRSA are consistent with reports from prospective surveillance studies showing declining MRSA rates in hospital settings. These studies attribute declining healthcare-associated MRSA to following implementation of IPC programs, active surveillance, decolonization strategies, and antimicrobial stewardship [19, 20]. The steeper MRSA decline in our study may reflect the impact of interventions especially targeting resistant strain or evolving specimen collection practices [21, 22], however, because our case-case-control design compares pathogens within a surveillance dataset rather than measuring population-level incidence or prevalence, causal conclusions cannot be drawn. These findings should be confirmed in prospective population-based studies.

The high odds of *S. aureus* isolation in school-age children and adolescents likely reflect high staphylococcal colonization rates and common skin and soft tissue infections in this age group [1]. The paradoxically lower odds in older adults compared to infants could be attributed to the fact that enterococcus infections are particularly common in elderly patients due to high rates of urinary catheterization [23], as evidenced by 38.5% of enterococcus isolates from patients aged ≥ 65 years versus 32.8% for MRSA. The elevated staphylococcal odds in emergency departments and general medicine units likely reflects patient mix and infection types, with these settings commonly evaluating skin, soft tissue, and respiratory infections where staphylococci predominate [24]. The specimen source findings of our study strongly align with known microbiology that *S. aureus* is a leading cause of pneumonia and skin infections versus enterococcus as a urinary and intra-abdominal pathogen, thereby supporting the validity of our approach.

The strong MRSA-levofloxacin co-resistance (OR = 11.19) findings of our study reflects well-established genetic linkages, as many prevalent MRSA clones carry both *SCCmec* elements which confers methicillin resistance and fluoroquinolone resistance mutation in *gyrA* and *grlA* [25, 26]. The substantially lower odds of resistance to linezolid, vancomycin, daptomycin, and other agents in *S. aureus* versus enterococcus reflects the high intrinsic and acquired resistance rate in enterococci [27]. These findings support continuous use of glycopeptides, oxazolidinones, and lipopeptides for serious staphylococcal infections, although judicious use remains critical to prevent resistance emergence.

The findings of this study suggest several IPC priorities. Countries and regions with elevated MRSA odds may benefit from enhanced prevention measures including contact precautions, active surveillance in high risks patients, decolonization protocols, environmental cleaning, and antimicrobial stewardship targeting fluoroquinolone use [28]. The high staphylococcal isolation odds from skin/soft tissue and respiratory sources reinforces established prevention strategies including surgical site IPC measure, ventilator-associated pneumonia prevention, and appropriate skin antisepsis [12, 20]. The strong methicillin-fluoroquinolone co-resistance supports guidelines recommending against empiric fluoroquinolone monotherapy in high-MRSA prevalence setting. IPC should be tailored to local epidemiology rather than applying uniform global strategies, with surveillance data informing region-specific approaches [29].

The analysis of this study demonstrates both the value and limitations of large-scale AMR surveillance. ATLAS provides a unique insight into resistance patterns across diverse settings and extended periods, yet convenience sampling, variable participation, and limited clinical data constrain causal inference. Future surveillance should strive for more representative sampling frameworks, standardized inclusion criteria, and collection of detailed clinical variables, including comorbidities, healthcare exposures, antimicrobial history, and infection syndrome classification. Integration of genomic surveillance with phenotypic data could help elucidate clonal dynamics, resistance evolution, and transmission patterns [30]. Comparative analysis with population-based studies would help calibrate the interpretation of our surveillance data and inform optimal sampling strategies. Linkage of resistance data with antimicrobial consumption, IPC practices, and healthcare system characteristics could enable ecological analyses of relationships between interventions and resistance trends moving forward [31].

## Conclusion

This multinational country-level surveillance analysis describes considerable geographic, temporal, and clinical variation in factors associated with methicillin-resistant *Staphylococcus aureus* and methicillin-susceptible *Staphylococcus aureus* isolation within a surveillance dataset. The findings of this study may support region-specific infection control strategies, continued antimicrobial stewardship, and ongoing global resistance surveillance. The persistent low resistance rates to glycopeptides and oxazolidinones in *S. aureus* are encouraging, while high fluoroquinolone co-resistance in MRSA underscores the need for judicious antimicrobial use. Large scale surveillance programs like ATLAS provide valuable data for monitoring resistance trends and informing public health priorities, though integration with detailed epidemiological and genomic data would enhance their utility in combating AMR.

## Supplementary Information

Below is the link to the electronic supplementary material.

Supplementary Material 1 (XLSX 34.3 KB)

## Data Availability

The data used for this study is readily available from Vivli Center for Global Clinical Research https://amr.vivli.org.
